# The missing spleen – A diagnosis of medical identity fraud in surgery: Case report

**DOI:** 10.1016/j.ijscr.2020.12.052

**Published:** 2020-12-24

**Authors:** Roy Huynh, Alexandra Thoms, Thuy-My Nguyen, Titus Kwok

**Affiliations:** a Department of Upper Gastrointestinal Surgery, Concord Repatriation General Hospital, Sydney, New South Wales, Australia; b Faculty of Medicine, University of New South Wales, Sydney, Australia

**Keywords:** Surgery, Endoscopy, Foreign body ingestion, Medical identity fraud, Medicolegal, Case report

## Abstract

**Introduction and importance:**

Medical identity fraud is a growing concern in surgery and can adversely affect patient care. Fraudulent medical information can result in misdiagnoses or inadequate preoperative workup of surgical patients.

**Case presentation:**

A 63-year-old female presented to hospital with a fishbone-related oesophageal perforation. Unbeknownst to the surgical team, the patient was committing medical identity fraud and impersonating another 57-year-old female. She underwent a gastroscopy with removal of the fishbone while her true identity remained concealed. A progress CT scan performed three days post-gastroscopy revealed an absent spleen, which was present on ultrasound as recent as three weeks prior to her admission. The patient was confronted about this discrepancy and confessed to committing medical identity fraud.

**Clinical discussion:**

Detection of medical identity fraud remains a challenging problem in healthcare systems worldwide. Various methods have been proposed to improve detection of medical identity fraud but there has been limited studies on their effectiveness in hospital settings. Given the serious health repercussions that can arise from medical identity fraud, it is imperative that better detection methods become available in the future.

**Conclusion:**

Medical identity fraud is a complex problem that requires better detection methods to prevent patient harm. Surgeons should be aware of the adverse health consequences that can arise from medical identity fraud.

## Highlights

• Medical identity fraud is a serious issue in surgery that is often under-reported.
• Serious surgical complications can arise because of medical identity fraud.
• The use of electronic medical records reduces the risk of medical identity fraud.
• There is a need for better detection of medical identity fraud in surgery.

## 1 Introduction

Medical identity fraud is a challenging problem in surgery and is known to cause adverse health outcomes [1]. The practice of medical identity fraud varies between countries due to differences in healthcare systems. In the United States, the financial loss incurred by the government was $14.1 billion, affecting an estimated 2.3 million victims in 2014 [2]. The scope of the problem is expected to grow with a lack of effective counteracting strategies [3]. In Australia, medical identity fraud is less of a problem due to centralization of a state medical electronic system that records patients’ previous hospital admissions. Any discrepancies in the patient’s history can be detected by clinicians reviewing their patient’s medical electronic record [4]. However, in patients with complex admissions, these discrepancies may not be picked up and potential medical identity fraud may occur undetected.

The potential health consequences of medical identity fraud are severe, particularly if surgery is involved. Incorrect medical information provided by the perpetrator can result in improper preoperative workup, misdiagnosis, and adverse patient outcomes [5]. Outside of the healthcare setting, medical identity fraud can result in life-altering circumstances such as being pursued by debt collectors of private medical insurance companies or having children taken away due to false information of drug abuse documented in their medical history [6]. Despite the seriousness of the problem, medical identity fraud is not commonly discussed in surgery and there has been limited documentation of its occurrence.

In this case report, we discuss a patient who underwent an endoscopic procedure while, unbeknownst to the treating team, was committing identity fraud. Her true identity was uncovered upon realizing discrepancies in her imaging studies. The potential health impact to the patient and the person she was impersonating are discussed in this paper. This case report has been reported in line with the SCARE Guidelines 2020 [7].

## 2 Case presentation

A 63-year-old female presented to the emergency department with throat pain following ingestion of a fish bone. Unbeknownst to any of the hospital staff, the patient was assuming the identity of another 57-year-old female. There was no suspicious behaviour at the time to suggest that the patient was committing identity fraud. She spoke an uncommon Chinese dialect and was accompanied by her niece who was able to assist minimally with English translation. The patient described odynophagia, which started immediately after accidental ingestion of a fishbone. She had a CT chest arranged by her GP prior to coming to hospital, which showed a 25 mm opaque foreign body in the upper oesophagus at the T2/3 level associated with a small, contained perforation.

Examination revealed a hemodynamically well patient with a midline laparotomy scar and mild epigastric tenderness. The patient was unable to provide a surgical history consistent with her midline laparotomy. This was assumed to be due to language barrier preventing accurate translation from the patient’s niece to the attending doctor. The patient’s electronic medical record was extensively reviewed and did not reveal any previous surgeries. It was noted however that she had a documented history of chronic hepatitis B and had been attending the hospital’s gastroenterology clinic with regular abdominal ultrasounds to assess her liver. Besides this, the patient did not have any other significant history recorded.

**Fig. 1:**
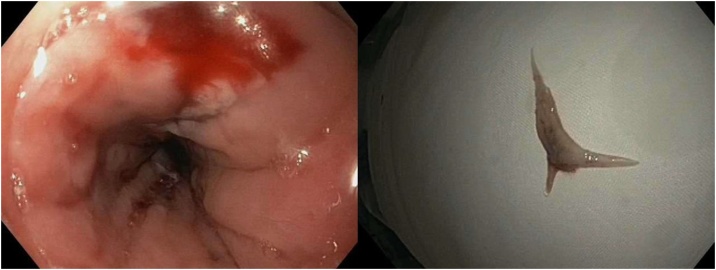
Endoscopic images showing the site of esophageal perforation (right) caused by ingested fishbone (left).

**Fig. 2:**
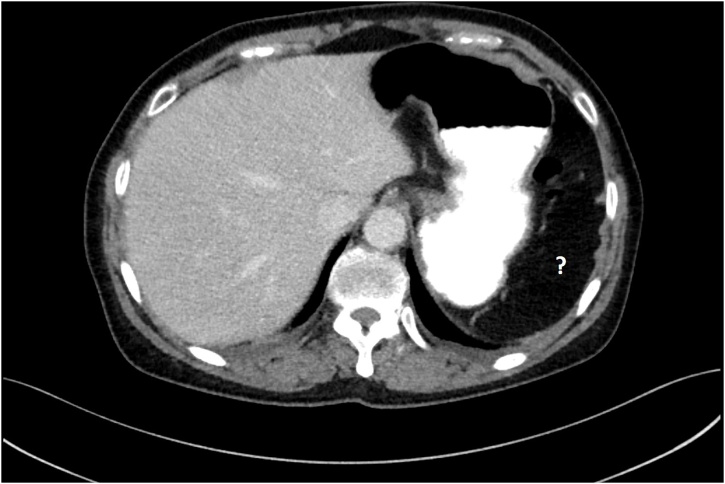
Transverse CT slice showing an absent spleen as denoted by a question mark.

**Fig. 3:**
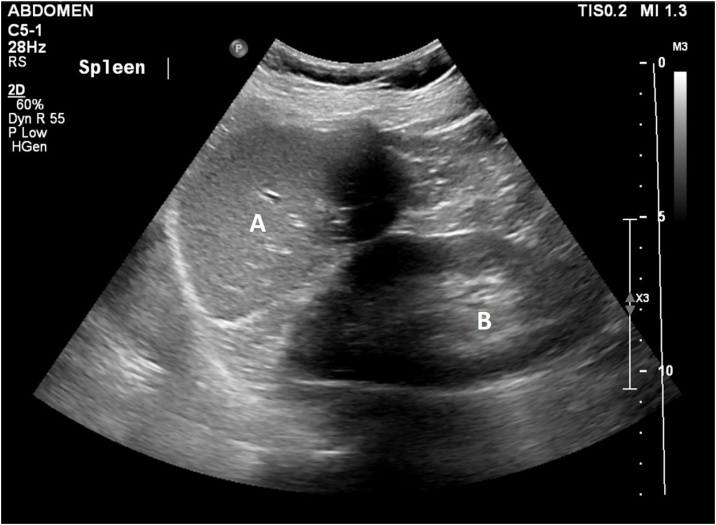
Abdominal ultrasound of left upper quadrant showing (A) spleen and (B) left kidney.

The patient underwent a gastroscopy where a foreign body resembling a fishbone was removed endoscopically by a surgeon (Fig. 1). She tolerated the procedure well and remained clinically stable post-procedurally. She was kept fasted for three days before a progress CT chest confirmed that the oesophageal perforation had healed. The CT chest incorporated the upper abdomen down to the L4 vertebra. The spleen was notably absent on the CT scan (Fig. 2), raising the possibility that the patient may have had an emergency splenectomy in the past, which would explain her midline laparotomy scar. However, her previous abdominal ultrasounds including one performed as recently as three weeks prior to her admission demonstrated that she still had her spleen (Fig. 3). This imaging discrepancy raised suspicion of possible medical identity fraud.

The patient was directly informed of the discrepancy seen in her imaging. She confessed to committing medical identity fraud, stating that she was an international visitor hoping to avoid the out-of-pocket cost associated with hospital admission. The seriousness of her actions was emphasized to her and she agreed to pay the full cost of her hospital stay. Following consultation with the hospital’s medicolegal department in conjunction with the victim of identity fraud, a decision was made not to press any charges. A telehealth follow-up appointment at four weeks demonstrated that the patient was at her baseline health. She was tolerating solid foods and denied having any chest pain. The patient expressed sincere regret at having committed identity fraud and provided a verbal apology to the hospital.

## 3 Discussion

Medical identity fraud enables imposters to obtain medical services that they would otherwise have to pay out-of-pocket or be ineligible for [8]. The impact of medical identity fraud is often reported in terms of financial costs with very little focus on patient care [9]. Patients who assume a false identity may undergo certain procedures without having adequate preoperative workup. In our case, the patient underwent a gastroscopy with key aspects of her past medical history concealed, including the fact she had a previous splenectomy. This information would have been important had she required more extensive surgery as it would allow the surgeons to anticipate and plan for a more difficult dissection. Furthermore, should a needlestick or splash injury have occurred during the patient’s admission, the documented history of hepatitis B on the patient’s electronic record may have caused undue stress to staff. Medical identity fraud also has serious repercussions to victims with misinformation imprinted on their medical record affecting their future healthcare. Our patient’s gastroscopy report was inscribed onto the medical record of the person she was impersonating. Had this remained undetected, it could potentially affect future clinical decisions regarding the victim’s health such as falsely reassuring clinicians of a normal stomach and duodenum if the victim were to present soon with epigastric pain and early satiety.

A centralised electronic medical record system reduces the risk of medical identity fraud by allowing clinicians to detect discrepancy between the patient’s presentation and past medical history [4]. However, this requires the clinicians to actively look for discrepancy, which is rarely done routinely given the low incidence of medical identity fraud. In our case, the discrepancy seen in our patient’s imaging was detected incidentally. There have been multiple proposals on various methods in improving detection of medical identity fraud. These range from designing better medical record systems to training hospital staff to become more familiar with medical identity theft policies [10,11]. Unfortunately, these approaches have yet been studied in a pragmatic setting to assess their effectiveness in mitigating medical identity fraud.

## 4 Conclusion

Our case highlights the potential health consequences that can arise when looking after a surgical patient who has committed medical identity fraud. Thankfully, our patient’s entire admission was not marred by any serious complications related to her fraudulent practice. However, this could have easily not being the case if the patient had a more complicated medical background or required more invasive interventions. Overall, medical identity fraud is a serious issue that demands better detection methods to avoid adverse health consequences associated with it. Surgeons should recognise that it is a complex problem that demands amplified awareness to prevent patient harm.

## Declaration of Competing Interest

The authors report no declarations of interest.

## Funding

This case report did not receive any funding.

## Ethical approval

This is a case report and is exempted from ethical approval at our institution.

## Consent

Written informed consent was obtained from the patient for publication of this case report and accompanying images. A copy of the written consent is available for review by the Editor-in-Chief of this journal on request.

Identifying details have been omitted from the text and from the figures/images.

## Author contribution

Dr Roy Huynh: Care of patient described in case report, conception and design of study, acquisition of data, analysis and interpretation of data, drafting the manuscript, revising the manuscript for important intellectual content, approval of final manuscript.

Dr Alexandra Thoms: Care of patient described in case report, conception and design of study, acquisition of data, analysis and interpretation of data, revising the manuscript for important intellectual content, approval of final manuscript.

Dr Thuy-My Nguyen: Care of patient described in case report, analysis and interpretation of data, revising the manuscript for important intellectual content, care of patient described in case report, advice and council on case report, approval of final manuscript.

Dr Titus Kwok: Care of patient described in case report, acquisition of data, analysis and interpretation of data, supervision of case report, revising the manuscript for important intellectual content, approval of final manuscript.

## Registration of research studies

Not applicable.

## Guarantor

Dr. Roy Huynh.

## Provenance and peer review

Not commissioned, externally peer-reviewed.
